# Supplementary material for: The risk factors associated with treatment-related mortality in 16,073 kidney transplantation—A nationwide cohort study
Source: PLoS One. 2020 Jul 28;15(7):e0236274. doi: 10.1371/journal.pone.0236274 (PMC7386583; doi:10.1371/journal.pone.0236274)
Supplement: Attachment — Submitted filename: Response to Reviewers.doc [file pone.0236274.s002.doc]

1. Thank you for providing the following Data Availability Statement:

The repository data for public release is not available because of the personally dentifiable information. The full dataset includes clinic centers in which they attend, insurance conditions. Therefore, concerning privacy risks, the data is managed by authorized executive supervisor. If one researcher asks to access data, the person in charge releases data with blind identification for the discrete requirements and the data should be analyzed only in permitted rooms in centers of National Health Insurance Service. Subsets of data limited to anonymisable information obtained and analyzed during this study are included in this article (tables, figures, and supporting information). Contact information for a data access committee is listed as follows: National Health Insurance Sharing Service, Tel: 82-33-736-2432; Official internet site: https://nhiss.nhis.or.kr/bd/ay/bdaya001iv.do."

Before we proceed, please confirm the following:

1) Please give the full name of the organization that has imposed the data restictions (e.g., a Research Ethics Committee or Institutional Review Board, etc.).

- We submitted the security memorandum and pledge to the Institutional Review Board of National Health Insurance Sharing Service when we access these data. The original files of security memorandum and pledge have been uploaded for this revision. The translated contents include “Any data obtained from National Health Insurance Sharing Service will not be taken out externally or used for any other purpose. I pledge to take any civil and criminal penalties.”. Your kind consideration for this security situation would be greatly appreciated. We have inserted additional explanation in to the revised Data Availability Statement (page 20, lines 11 to 13) as follows.

“If one researcher asks to access data, the researcher should submit the security memorandum and pledge to the Institutional Review Board of National Health Insurance Sharing Service. After approval, the person in charge releases data with blind identification for the discrete requirements and the data should be analyzed only in permitted rooms in centers of National Health Insurance Service.”

2) Please confirm that others would be able to access these data in the same manner as the authors. Please also confirm that the authors did not have any special access privileges that others would not have.

- We confirmed that others could access these data in the same manner as the authors and the authors did not have any special access privileges. We also have added these statement to the revised Data Availability Statement (page 20, lines 15 to 16) as follows.

“The other researchers could access these data in the same manner as the authors and the authors did not have any special access privileges.”

3) Please confirm that the data that researchers can access fits our definition of "minimal data set" as outlined here: https://journals.plos.org/plosone/s/data-availability#loc-minimal-data-set-definition

**🡪** We have provided maximally permitted data for meeting the requirements of “minimal data set”. Although entire data sets of kidney recipients were not permitted, the anonymisable data for recipients with treatment-related mortality focused on this manuscript and used for tables and graphs were provided in S1 Table.

**Journal requirements:**

1. Please ensure that your manuscript meets PLOS ONE's style requirements, including those for file naming. The PLOS ONE style templates can be found at http://www.plosone.org/attachments/PLOSOne_formatting_sample_main_body.pdf and http://www.plosone.org/attachments/PLOSOne_formatting_sample_title_authors_affiliations.pdf

- We have checked the PLOS ONE style templates and corrected the location of References section. The revised References section has been listed after the main text, before the supporting information. The file naming also checked and corrected.

2. We note that you have indicated that data from this study are available upon request. PLOS only allows data to be available upon request if there are legal or ethical restrictions on sharing data publicly. For information on unacceptable data access restrictions, please see http://journals.plos.org/plosone/s/data-availability#loc-unacceptable-data-access-restrictions.

In your revised cover letter, please address the following prompts:

a) If there are ethical or legal restrictions on sharing a de-identified data set, please explain them in detail (e.g., data contain potentially identifying or sensitive patient information) and who has imposed them (e.g., an ethics committee). Please also provide contact information for a data access committee, ethics committee, or other institutional body to which data requests may be sent.

b) If there are no restrictions, please upload the minimal anonymized data set necessary to replicate your study findings as either Supporting Information files or to a stable, public repository and provide us with the relevant URLs, DOIs, or accession numbers. Please see http://www.bmj.com/content/340/bmj.c181.long for guidelines on how to de-identify and prepare clinical data for publication. For a list of acceptable repositories, please see http://journals.plos.org/plosone/s/data-availability#loc-recommended-repositories.

We will update your Data Availability statement on your behalf to reflect the information you provide.

- We provided the minimal data set, which had anonymisable information in S1 Table. Therefore, we have corrected the sentence from “Subsets of data limited to anonymisable information obtained and analyzed during this study are included in this article (tables, figures, and supporting information) and are available from the corresponding author upon reasonable request.” to “Subsets of data limited to anonymisable information obtained and analyzed during this study are included in this article (tables, figures, and supporting information).” in the revised Data accessibility statement section (page 20, lines 17 to 18). Because National Health Insurance Sharing Service restricts to share full dataset concerning privacy risks, minimal anonymized data set focusing on recipients with treatment-related mortality after kidney transplantation was provided. Contact information for a data access committee and the way for obtaining the data were described in the Data accessibility statement section. We have added these statement to the revised cover letter.

**Response to the reviewer’s comments**

1. **Is the manuscript technically sound, and do the data support the conclusions?**

**Reviewer #1: Partly**

- We have corrected and checked that the revised manuscript described a technically sound piece of scientific research and that the data supported the conclusions. We also responded to the reviewer #1’s comments sincerely.

**2. Has the statistical analysis been performed appropriately and rigorously?**

**Reviewer #1: Yes**

- We have checked that the statistical analysis has been conducted appropriately and rigorously.

**3. Have the authors made all data underlying the findings in their manuscript fully available?**

**Reviewer #1: Yes**

- According to the journal requirements, we provided the minimal data set focusing on recipients with treatment-related mortality after kidney transplantation. Contact information for a data access committee and the way for obtaining the data were described in the Data accessibility statement section. We have added these statement to the revised cover letter.

**“**We collected the following demographic data and baseline characteristics of kidney recipients from the HIRA database: age, sex, medical comorbidities focusing on cardiac and cerebrovascular diseases reported to be important causes of early mortality [16], dialysis status, cytomegalovirus (CMV) and fungal infection, and year of transplantation (S1 Table).”

**S1 Table. Data set of recipients with TRM after kidney transplantation.**

**4. Is the manuscript presented in an intelligible fashion and written in standard English?**

**Reviewer #1: Yes**

- We used manuscript editing service before submission.

5. **Review Comments to the Author**

Please use the space provided to explain your answers to the questions above. You may also include additional comments for the author, including concerns about dual publication, research ethics, or publication ethics. (Please upload your review as an attachment if it exceeds 20,000 characters)

**Response to reviewer #1’s comments**

**Comment 1**: In this paper submitted by Jeong et al, an investigation about the risk factors causes of TRM after kidney transplantation, focusing on vascular diseases was presented.

The paper is written in a correct and fluent English, and statistical analyses are correctly described and presented by the authors.

Nevertheless, the paper presents several limitations, apart the retrospective design, which make oit unsuitable for the publication in this form. First of all, several important data are missing and in my opinion crucial for the aim of the study: dialysis vintage, prevalence of deceased/living donor (if not considered explain why), basic nephropathy, and steroid therapy before therapy, donor characteristics. All those factors might impact also on the global results of the study that at the moment does not add any novel knowledge on the problem.

**🡪 Response 1**: As indicated by reviewer, we have provided available information in the revised manuscript.

**Dialysis vintage:**

We have requested additional data from another database operated by the Korean Network for Organ Sharing system in order to analyze dialysis vintage. In this database, the records of recipients who underwent kidney transplantation in 40 medical centers around the country have been registered. We have provided the results of dialysis vintage, time from dialysis to transplantation, in the revised Tables 1-3 as follows. The source of data was also described in revised Materials and Methods section as follows.

**Results**

Table 1. Comparison of the characteristics between living kidney recipients versus deceased ones at 1 and 3 months after transplantation.

| **Characteristics^a^** | **Early TRM** | | | **TRM** | | |
| --- | --- | --- | --- | --- | --- | --- |
|  | **Living at 1 month** | **Death by 1 month** | ***P*-value^b^** | **Living at 3 months** | **Death by 3 months** | ***P*-value^b^** |
| Number (%) | 15,999 | 74 |  | 15,913 | 160 |  |
| Age, years | 47 (37-55) | 56 (48.8-61) | < 0.001 | 47 (37-55) | 55.5 (48-61) | < 0.001 |
| < 50 | 9,188 (57.4) | 19 (25.7) | < 0.001 | 9,163 (57.6) | 44 (27.5) | < 0.001 |
| 50–59 | 4,830 (30.2) | 32 (43.2) |  | 4,792 (30.1) | 70 (43.8) |  |
| 60–69 | 1,838 (11.5) | 18 (24.3) |  | 1,819 (11.4) | 37 (23.1) |  |
| 70–79 | 143 (0.9) | 5 (6.8) |  | 139 (0.9) | 9 (5.6) |  |
| Sex, male | 9,451 (59.1) | 44 (59.5) | 0.946 | 9,403 (59.1) | 92 (57.5) | 0.684 |
| Cause of ESRD |  |  |  |  |  |  |
| Diabetes mellitus | 3,501 (21.9) | 19 (25.7) | 0.431 | 3,479 (21.9) | 41 (25.6) | 0.252 |
| Hypertension | 2,001 (12.5) | 8 (10.8) | 0.660 | 1,992 (12.5) | 17 (10.6) | 0.471 |
| Glomerulonephritis | 2,850 (17.8) | 11 (14.9) | 0.508 | 2,840 (17.8) | 21 (13.1) | 0.120 |
| Cystic kidney disease | 368 (2.3) | 2 (2.7) | 0.818 | 365 (2.3) | 5 (3.1) | 0.485 |
| Underlying disease^c^ |  |  |  |  |  |  |
| Cardiac disease |  |  |  |  |  |  |
| Coronary artery disease | 392 (2.5) | 9 (12.2) | < 0.001 | 384 (2.4) | 17 (10.6) | < 0.001 |
| Acute myocardial infarction | 288 (1.8) | 2 (2.7) | 0.561 | 283 (1.8) | 7 (4.4) | 0.014 |
| Cardiac arrhythmia | 1,240 (7.8) | 13 (17.6) | 0.002 | 1,230 (7.7) | 23 (14.4) | 0.002 |
| Cerebrovascular disease |  |  |  |  |  |  |
| Cerebral hemorrhage | 54 (0.3) | 0 (0.0) | 0.617 | 54 (0.3) | 0 (0.0) | 0.460 |
| Cerebral infarction | 247 (1.5) | 1 (1.4) | 0.893 | 246 (1.5) | 2 (1.3) | 0.763 |
| Hemodialysis | 13,134 (82.1) | 69 (93.2) | 0.012 | 13,055 (82.0) | 148 (92.5) | 0.001 |
| Dialysis vintage, months^d^ | 42.5 (29.5-62.8) | 16.0 (9.5-24.5) | 0.051 | 41.0 (29.0-63.5) | 24.5 (12.8-39.0) | 0.179 |
| Before steroid use^e^ | 1,148 (7.2) | 3 (4.1) | 0.416 | 1,140 (7.2) | 11 (6.9) | 1.000 |
| Infection |  |  |  |  |  |  |
| CMV infection | 694 (4.3) | 4 (5.4) | 0.653 | 1,900 (11.9) | 37 (23.1) | < 0.001 |
| Fungal infection | 639 (4.0) | 2 (2.7) | 0.571 | 1,205 (7.6) | 37 (23.1) | < 0.001 |
| Epoch of transplantation |  |  |  |  |  |  |
| 2003–2009 | 4,634 (29.0) | 27 (36.5) | 0.155 | 4,594 (28.9) | 67 (41.9) | < 0.001 |
| 2010–2016 | 11,365 (71.0) | 47 (63.5) |  | 11,319 (71.1) | 93 (58.1) |  |

^a^ Data were expressed as number (%) or median (interquartile range).

^b^ *P* value was calculated using chi-square test or Mann–Whitney U test.

^c^ In case of the presence of underlying diseases, multiple diseases were designated to one patient.

^d^ Data were obtained from the Korean Network for Organ Sharing system.

^e^ The use of intravenous steroids such as dexamethasone, and prednisolone within 6 months before transplantation.

Abbreviations: CMV, cytomegalovirus; ESRD, end-stage renal disease; TRM, treatment-related mortality.

Table 2. Univariate and multivariate analyses of 1-month mortality after kidney transplantation.

| Variable | Univariate | | Multivariate | |
| --- | --- | --- | --- | --- |
|  | HR (95% CI) | *P*-value | HR (95% CI) | *P*-value |
| Age, years^a^ | 1.07 (1.05–1.10) | < 0.001 | 1.07 (1.05-1.10) | < 0.001 |
| < 50 | Reference |  |  |  |
| 50–59 | 3.21 (1.82-5.66) | < 0.001 |  |  |
| 60–69 | 4.74 (2.49-9.03) | < 0.001 |  |  |
| 70–79 | 16.66 (6.22-44.62) | < 0.001 |  |  |
| Sex, male | 0.98 (0.62-1.56) | 0.944 |  |  |
| Cause of ESRD |  |  |  |  |
| Diabetes mellitus | 1.22 (0.89-1.75) | 0.451 |  |  |
| Hypertension | 0.80 (0.58-1.12) | 0.672 |  |  |
| Glomerulonephritis | 0.93 (0.52-2.15) | 0.591 |  |  |
| Cystic kidney disease | 1.19 (0.78-2.32) | 0.854 |  |  |
| Underlying disease |  |  |  |  |
| Cardiac disease |  |  |  |  |
| Coronary artery disease | 5.51 (2.74-11.06) | < 0.001 | 2.81 (1.37-5.78) | 0.005 |
| Acute myocardial infarction | 1.51 (0.37-6.15) | 0.566 |  |  |
| Cardiac arrhythmia | 2.53 (1.39-4.60) | 0.002 | 1.99 (1.09-3.64) | 0.025 |
| Cerebrovascular disease |  |  |  |  |
| Cerebral hemorrhage | NA |  |  |  |
| Cerebral infarction | 0.87 (0.12-6.26) | 0.890 |  |  |
| Hemodialysis | 3.00 (1.21-7.45) | 0.018 | 2.58 (1.04-6.42) | 0.041 |
| Dialysis vintage, months^c^ | 0.918 (0.833-1.012) | 0.086 |  |  |
| Before steroid use^d^ | 0.55 (0.17-1.74) | 0.307 |  |  |
| Infection |  |  |  |  |
| CMV infection | 1.26 (0.46-3.45) | 0.652 |  |  |
| Fungal infection | 0.66 (0.16-2.71) | 0.569 |  |  |
| Epoch of transplantation, 2010–2016 | 0.72 (0.45-1.15) | 0.168 |  |  |

^a^ Variables less than 0.05 of *P*-values in univariate analysis were included in the multivariate analysis.

^b^ NA is presented if the paucity of deceased or living patients exists for each variable 1 month after kidney transplantation.

^c^ Data were obtained from the Korean Network for Organ Sharing system.

^d^ The use of intravenous steroids such as dexamethasone, and prednisolone within 6 months before transplantation.Abbreviations: CI, confidence interval; CMV, cytomegalovirus; ESRD, end-stage renal disease; HR, hazard ratio; NA, not applicable.

**Table 3. Univariate and multivariate analyses of 3-month mortality after kidney** transplantation.

| Variable | Univariate | | Multivariate | |
| --- | --- | --- | --- | --- |
|  | HR (95% CI) | *P*-value | HR (95% CI) | *P*-value |
| Age, years^a^ | 1.07 (1.05–1.09) | < 0.001 | 1.08 (1.06-1.09) | < 0.001 |
| < 50 |  |  |  |  |
| 50–59 | 3.05 (2.09-4.44) | < 0.001 |  |  |
| 60–69 | 4.24 (2.74-6.57) | < 0.001 |  |  |
| 70–79 | 13.16 (6.43-26.96) | < 0.001 |  |  |
| Sex, female | 1.07 (0.78-1.46) | 0.690 |  |  |
| Cause of ESRD |  |  |  |  |
| Diabetes mellitus | 1.25 (0.92-1.59) | 0.273 |  |  |
| Hypertension | 0.86 (0.68-1.10) | 0.463 |  |  |
| Glomerulonephritis | 0.91 (0.49-1.75) | 0.385 |  |  |
| Cystic kidney disease | 1.23 (0.87-2.41) | 0.526 |  |  |
| Underlying disease |  |  |  |  |
| Cardiac disease |  |  |  |  |
| Coronary artery disease | 4.82 (2.92-7.97) | < 0.001 | 2.53 (1.49-4.31) | 0.001 |
| Acute myocardial infarction | 2.48 (1.16-5.29) | 0.019 | 1.70 (0.78-3.69) | 0.183 |
| Cardiac arrhythmia | 1.99 (1.28-3.10) | 0.002 | 1.43 (0.91-2.24) | 0.117 |
| Cerebrovascular disease |  |  |  |  |
| Cerebral hemorrhage | NA |  |  |  |
| Cerebral infarction | 0.80 (0.20-3.23) | 0.755 |  |  |
| Hemodialysis | 2.69 (1.49-4.85) | 0.001 | 2.32 (1.28-4.19) | 0.005 |
| Dialysis vintage, months^c^ | 0.963 (0.911-1.017) | 0.179 |  |  |
| Before steroid use^d^ | 0.95 (0.52-1.76) | 0.882 |  |  |
| Infection |  |  |  |  |
| CMV infection | 2.19 (1.51-3.16) | < 0.001 | 1.65 (1.12-2.42) | 0.012 |
| Fungal infection | 3.57 (2.47-5.15) | < 0.001 | 2.48 (1.69-3.65) | < 0.001 |
| Epoch of transplantation, 2010–2016 | 0.58 (0.42-0.79) | 0.001 | 0.43 (0.31-0.60) | < 0.001 |

^a^ Variables less than 0.05 of *P*-values in univariate analysis were included in the multivariate analysis.

^b^ NA is presented if the paucity of deceased or living patients exists for each variable 3 months after kidney transplantation.

^c^ Data were obtained from the Korean Network for Organ Sharing system.

^d^ The use of intravenous steroids such as dexamethasone, and prednisolone within 6 months before transplantation.

Abbreviations: CI, confidence interval; CMV, cytomegalovirus; ESRD, end-stage renal disease; HR, hazard ratio; NA, not applicable.

**Materials and Methods section** (page 7, lines 11 to 14)

The data for dialysis vintage, and donor state omitting in HIRA database were obtained from another database operated by the Korean Network for Organ Sharing system. In this database, the records of recipients who underwent kidney transplantation in 40 medical centers around the country were registered.

**Prevalence of deceased/living donor (if not considered explain why):**

We have also requested data of donor state from the Korean Network for Organ Sharing system since donor information was not included in the Health Insurance Review and Assessment Service (HIRA). The results of living, deceased, and non-heart beating donors are presented in the revised Results section as follows. These variables did not insert into the Tables because the direct combination of data was not available for the multivariate analyses.

**Results**

**Characteristics of patients** (page 8, lines 11 to 13)

“Most patients received kidney from living donor (62.2%), followed by deceased (37.5%) and non-heart beating (0.3%) donors.”

**Treatment-related mortality** (page 11, lines 9 to 12)

“The rates of recipients who died 1 month (1.2% for living, 2.4% for deceased, and 3.3% for non-heart beating) and 3 months (3.2% for living, 5.5% for deceased, and 3.3% for non-heart beating) after transplantation showed significant difference according to the donor state (*P* < 0.001).”

**Discussion** (page 17, lines 22 to page 18 lines 3)

“Donor status has been a well-known important factor for short- and long-term mortality after kidney transplantation [15,21]. According to previous studies, kidney allograft recipients that died within the first year after transplantation were more likely to be recipients of deceased donor kidneys [18,20]. It was difficult to compare TRM of our cohort with those of other countries directly because of lack of available data. More intensive care for recipients from deceased donors at early point after transplantation is recommended.”

**Basic nephropathy:**

We have added the results of basic nephropathy including diabetes mellitus, hypertension, glomerulonephritis, and cystic kidney disease to the revised Tables 1-3 as follows.

Table 1. Comparison of the characteristics between living kidney recipients versus deceased ones at 1 and 3 months after transplantation.

| **Characteristics^a^** | **Early TRM** | | | **TRM** | | |
| --- | --- | --- | --- | --- | --- | --- |
|  | **Living at 1 month** | **Death by 1 month** | ***P*-value^b^** | **Living at 3 months** | **Death by 3 months** | ***P*-value^b^** |
| Number (%) | 15,999 | 74 |  | 15,913 | 160 |  |
| Age, years | 47 (37-55) | 56 (48.8-61) | < 0.001 | 47 (37-55) | 55.5 (48-61) | < 0.001 |
| < 50 | 9,188 (57.4) | 19 (25.7) | < 0.001 | 9,163 (57.6) | 44 (27.5) | < 0.001 |
| 50–59 | 4,830 (30.2) | 32 (43.2) |  | 4,792 (30.1) | 70 (43.8) |  |
| 60–69 | 1,838 (11.5) | 18 (24.3) |  | 1,819 (11.4) | 37 (23.1) |  |
| 70–79 | 143 (0.9) | 5 (6.8) |  | 139 (0.9) | 9 (5.6) |  |
| Sex, male | 9,451 (59.1) | 44 (59.5) | 0.946 | 9,403 (59.1) | 92 (57.5) | 0.684 |
| Cause of ESRD |  |  |  |  |  |  |
| Diabetes mellitus | 3,501 (21.9) | 19 (25.7) | 0.431 | 3,479 (21.9) | 41 (25.6) | 0.252 |
| Hypertension | 2,001 (12.5) | 8 (10.8) | 0.660 | 1,992 (12.5) | 17 (10.6) | 0.471 |
| Glomerulonephritis | 2,850 (17.8) | 11 (14.9) | 0.508 | 2,840 (17.8) | 21 (13.1) | 0.120 |
| Cystic kidney disease | 368 (2.3) | 2 (2.7) | 0.818 | 365 (2.3) | 5 (3.1) | 0.485 |
| Underlying disease^c^ |  |  |  |  |  |  |
| Cardiac disease |  |  |  |  |  |  |
| Coronary artery disease | 392 (2.5) | 9 (12.2) | < 0.001 | 384 (2.4) | 17 (10.6) | < 0.001 |
| Acute myocardial infarction | 288 (1.8) | 2 (2.7) | 0.561 | 283 (1.8) | 7 (4.4) | 0.014 |
| Cardiac arrhythmia | 1,240 (7.8) | 13 (17.6) | 0.002 | 1,230 (7.7) | 23 (14.4) | 0.002 |
| Cerebrovascular disease |  |  |  |  |  |  |
| Cerebral hemorrhage | 54 (0.3) | 0 (0.0) | 0.617 | 54 (0.3) | 0 (0.0) | 0.460 |
| Cerebral infarction | 247 (1.5) | 1 (1.4) | 0.893 | 246 (1.5) | 2 (1.3) | 0.763 |
| Hemodialysis | 13,134 (82.1) | 69 (93.2) | 0.012 | 13,055 (82.0) | 148 (92.5) | 0.001 |
| Dialysis vintage, months^d^ | 42.5 (29.5-62.8) | 16.0 (9.5-24.5) | 0.051 | 41.0 (29.0-63.5) | 24.5 (12.8-39.0) | 0.179 |
| Before steroid use^e^ | 1,148 (7.2) | 3 (4.1) | 0.416 | 1,140 (7.2) | 11 (6.9) | 1.000 |
| Infection |  |  |  |  |  |  |
| CMV infection | 694 (4.3) | 4 (5.4) | 0.653 | 1,900 (11.9) | 37 (23.1) | < 0.001 |
| Fungal infection | 639 (4.0) | 2 (2.7) | 0.571 | 1,205 (7.6) | 37 (23.1) | < 0.001 |
| Epoch of transplantation |  |  |  |  |  |  |
| 2003–2009 | 4,634 (29.0) | 27 (36.5) | 0.155 | 4,594 (28.9) | 67 (41.9) | < 0.001 |
| 2010–2016 | 11,365 (71.0) | 47 (63.5) |  | 11,319 (71.1) | 93 (58.1) |  |

^a^ Data were expressed as number (%) or median (interquartile range).

^b^ *P* value was calculated using chi-square test or Mann–Whitney U test.

^c^ In case of the presence of underlying diseases, multiple diseases were designated to one patient.

^d^ Data were obtained from the Korean Network for Organ Sharing system.

^e^ The use of intravenous steroids such as dexamethasone, and prednisolone within 6 months before transplantation.

Abbreviations: CMV, cytomegalovirus; ESRD, end-stage renal disease; TRM, treatment-related mortality.

Table 2. Univariate and multivariate analyses of 1-month mortality after kidney transplantation.

| Variable | Univariate | | Multivariate | |
| --- | --- | --- | --- | --- |
|  | HR (95% CI) | *P*-value | HR (95% CI) | *P*-value |
| Age, years^a^ | 1.07 (1.05–1.10) | < 0.001 | 1.07 (1.05-1.10) | < 0.001 |
| < 50 | Reference |  |  |  |
| 50–59 | 3.21 (1.82-5.66) | < 0.001 |  |  |
| 60–69 | 4.74 (2.49-9.03) | < 0.001 |  |  |
| 70–79 | 16.66 (6.22-44.62) | < 0.001 |  |  |
| Sex, male | 0.98 (0.62-1.56) | 0.944 |  |  |
| Cause of ESRD |  |  |  |  |
| Diabetes mellitus | 1.22 (0.89-1.75) | 0.451 |  |  |
| Hypertension | 0.80 (0.58-1.12) | 0.672 |  |  |
| Glomerulonephritis | 0.93 (0.52-2.15) | 0.591 |  |  |
| Cystic kidney disease | 1.19 (0.78-2.32) | 0.854 |  |  |
| Underlying disease |  |  |  |  |
| Cardiac disease |  |  |  |  |
| Coronary artery disease | 5.51 (2.74-11.06) | < 0.001 | 2.81 (1.37-5.78) | 0.005 |
| Acute myocardial infarction | 1.51 (0.37-6.15) | 0.566 |  |  |
| Cardiac arrhythmia | 2.53 (1.39-4.60) | 0.002 | 1.99 (1.09-3.64) | 0.025 |
| Cerebrovascular disease |  |  |  |  |
| Cerebral hemorrhage | NA |  |  |  |
| Cerebral infarction | 0.87 (0.12-6.26) | 0.890 |  |  |
| Hemodialysis | 3.00 (1.21-7.45) | 0.018 | 2.58 (1.04-6.42) | 0.041 |
| Dialysis vintage, months^c^ | 0.918 (0.833-1.012) | 0.086 |  |  |
| Before steroid use^d^ | 0.55 (0.17-1.74) | 0.307 |  |  |
| Infection |  |  |  |  |
| CMV infection | 1.26 (0.46-3.45) | 0.652 |  |  |
| Fungal infection | 0.66 (0.16-2.71) | 0.569 |  |  |
| Epoch of transplantation, 2010–2016 | 0.72 (0.45-1.15) | 0.168 |  |  |

^a^ Variables less than 0.05 of *P*-values in univariate analysis were included in the multivariate analysis.

^b^ NA is presented if the paucity of deceased or living patients exists for each variable 1 month after kidney transplantation.

^c^ Data were obtained from the Korean Network for Organ Sharing system.

^d^ The use of intravenous steroids such as dexamethasone, and prednisolone within 6 months before transplantation.Abbreviations: CI, confidence interval; CMV, cytomegalovirus; ESRD, end-stage renal disease; HR, hazard ratio; NA, not applicable.

Table 3. Univariate and multivariate analyses of 3-month mortality after kidney transplantation.

| Variable | Univariate | | Multivariate | |
| --- | --- | --- | --- | --- |
|  | HR (95% CI) | *P*-value | HR (95% CI) | *P*-value |
| Age, years^a^ | 1.07 (1.05–1.09) | < 0.001 | 1.08 (1.06-1.09) | < 0.001 |
| < 50 |  |  |  |  |
| 50–59 | 3.05 (2.09-4.44) | < 0.001 |  |  |
| 60–69 | 4.24 (2.74-6.57) | < 0.001 |  |  |
| 70–79 | 13.16 (6.43-26.96) | < 0.001 |  |  |
| Sex, female | 1.07 (0.78-1.46) | 0.690 |  |  |
| Cause of ESRD |  |  |  |  |
| Diabetes mellitus | 1.25 (0.92-1.59) | 0.273 |  |  |
| Hypertension | 0.86 (0.68-1.10) | 0.463 |  |  |
| Glomerulonephritis | 0.91 (0.49-1.75) | 0.385 |  |  |
| Cystic kidney disease | 1.23 (0.87-2.41) | 0.526 |  |  |
| Underlying disease |  |  |  |  |
| Cardiac disease |  |  |  |  |
| Coronary artery disease | 4.82 (2.92-7.97) | < 0.001 | 2.53 (1.49-4.31) | 0.001 |
| Acute myocardial infarction | 2.48 (1.16-5.29) | 0.019 | 1.70 (0.78-3.69) | 0.183 |
| Cardiac arrhythmia | 1.99 (1.28-3.10) | 0.002 | 1.43 (0.91-2.24) | 0.117 |
| Cerebrovascular disease |  |  |  |  |
| Cerebral hemorrhage | NA |  |  |  |
| Cerebral infarction | 0.80 (0.20-3.23) | 0.755 |  |  |
| Hemodialysis | 2.69 (1.49-4.85) | 0.001 | 2.32 (1.28-4.19) | 0.005 |
| Dialysis vintage, months^c^ | 0.963 (0.911-1.017) | 0.179 |  |  |
| Before steroid use^d^ | 0.95 (0.52-1.76) | 0.882 |  |  |
| Infection |  |  |  |  |
| CMV infection | 2.19 (1.51-3.16) | < 0.001 | 1.65 (1.12-2.42) | 0.012 |
| Fungal infection | 3.57 (2.47-5.15) | < 0.001 | 2.48 (1.69-3.65) | < 0.001 |
| Epoch of transplantation, 2010–2016 | 0.58 (0.42-0.79) | 0.001 | 0.43 (0.31-0.60) | < 0.001 |

^a^ Variables less than 0.05 of *P*-values in univariate analysis were included in the multivariate analysis.

^b^ NA is presented if the paucity of deceased or living patients exists for each variable 3 months after kidney transplantation.

^c^ Data were obtained from the Korean Network for Organ Sharing system.

^d^ The use of intravenous steroids such as dexamethasone, and prednisolone within 6 months before transplantation.

Abbreviations: CI, confidence interval; CMV, cytomegalovirus; ESRD, end-stage renal disease; HR, hazard ratio; NA, not applicable.

**Steroid therapy before therapy:**

We have inserted the results of steroid therapy before transplantation into the revised Tables 1-3 as follows. The recipients with the use of intravenous steroids such as dexamethasone, and prednisolone within 6 months before transplantation were designated and described in the revised footnotes of Tables.

**Table 1. Comparison of the characteristics between living kidney recipients versus decea**sed ones at 1 and 3 months after transplantation.

| **Characteristics^a^** | **Early TRM** | | | **TRM** | | |
| --- | --- | --- | --- | --- | --- | --- |
|  | **Living at 1 month** | **Death by 1 month** | ***P*-value^b^** | **Living at 3 months** | **Death by 3 months** | ***P*-value^b^** |
| Number (%) | 15,999 | 74 |  | 15,913 | 160 |  |
| Age, years | 47 (37-55) | 56 (48.8-61) | < 0.001 | 47 (37-55) | 55.5 (48-61) | < 0.001 |
| < 50 | 9,188 (57.4) | 19 (25.7) | < 0.001 | 9,163 (57.6) | 44 (27.5) | < 0.001 |
| 50–59 | 4,830 (30.2) | 32 (43.2) |  | 4,792 (30.1) | 70 (43.8) |  |
| 60–69 | 1,838 (11.5) | 18 (24.3) |  | 1,819 (11.4) | 37 (23.1) |  |
| 70–79 | 143 (0.9) | 5 (6.8) |  | 139 (0.9) | 9 (5.6) |  |
| Sex, male | 9,451 (59.1) | 44 (59.5) | 0.946 | 9,403 (59.1) | 92 (57.5) | 0.684 |
| Cause of ESRD |  |  |  |  |  |  |
| Diabetes mellitus | 3,501 (21.9) | 19 (25.7) | 0.431 | 3,479 (21.9) | 41 (25.6) | 0.252 |
| Hypertension | 2,001 (12.5) | 8 (10.8) | 0.660 | 1,992 (12.5) | 17 (10.6) | 0.471 |
| Glomerulonephritis | 2,850 (17.8) | 11 (14.9) | 0.508 | 2,840 (17.8) | 21 (13.1) | 0.120 |
| Cystic kidney disease | 368 (2.3) | 2 (2.7) | 0.818 | 365 (2.3) | 5 (3.1) | 0.485 |
| Underlying disease^c^ |  |  |  |  |  |  |
| Cardiac disease |  |  |  |  |  |  |
| Coronary artery disease | 392 (2.5) | 9 (12.2) | < 0.001 | 384 (2.4) | 17 (10.6) | < 0.001 |
| Acute myocardial infarction | 288 (1.8) | 2 (2.7) | 0.561 | 283 (1.8) | 7 (4.4) | 0.014 |
| Cardiac arrhythmia | 1,240 (7.8) | 13 (17.6) | 0.002 | 1,230 (7.7) | 23 (14.4) | 0.002 |
| Cerebrovascular disease |  |  |  |  |  |  |
| Cerebral hemorrhage | 54 (0.3) | 0 (0.0) | 0.617 | 54 (0.3) | 0 (0.0) | 0.460 |
| Cerebral infarction | 247 (1.5) | 1 (1.4) | 0.893 | 246 (1.5) | 2 (1.3) | 0.763 |
| Hemodialysis | 13,134 (82.1) | 69 (93.2) | 0.012 | 13,055 (82.0) | 148 (92.5) | 0.001 |
| Dialysis vintage, months^d^ | 42.5 (29.5-62.8) | 16.0 (9.5-24.5) | 0.051 | 41.0 (29.0-63.5) | 24.5 (12.8-39.0) | 0.179 |
| Before steroid use^e^ | 1,148 (7.2) | 3 (4.1) | 0.416 | 1,140 (7.2) | 11 (6.9) | 1.000 |
| Infection |  |  |  |  |  |  |
| CMV infection | 694 (4.3) | 4 (5.4) | 0.653 | 1,900 (11.9) | 37 (23.1) | < 0.001 |
| Fungal infection | 639 (4.0) | 2 (2.7) | 0.571 | 1,205 (7.6) | 37 (23.1) | < 0.001 |
| Epoch of transplantation |  |  |  |  |  |  |
| 2003–2009 | 4,634 (29.0) | 27 (36.5) | 0.155 | 4,594 (28.9) | 67 (41.9) | < 0.001 |
| 2010–2016 | 11,365 (71.0) | 47 (63.5) |  | 11,319 (71.1) | 93 (58.1) |  |

^a^ Data were expressed as number (%) or median (interquartile range).

^b^ *P* value was calculated using chi-square test or Mann–Whitney U test.

^c^ In case of the presence of underlying diseases, multiple diseases were designated to one patient.

^d^ Data were obtained from the Korean Network for Organ Sharing system.

^e^ The use of intravenous steroids such as dexamethasone, and prednisolone within 6 months before transplantation.

Abbreviations: CMV, cytomegalovirus; ESRD, end-stage renal disease; TRM, treatment-related mortality.

Table 2. Univariate and multivariate analyses of 1-month mortality after kidney transplantation.

| Variable | Univariate | | Multivariate | |
| --- | --- | --- | --- | --- |
|  | HR (95% CI) | *P*-value | HR (95% CI) | *P*-value |
| Age, years^a^ | 1.07 (1.05–1.10) | < 0.001 | 1.07 (1.05-1.10) | < 0.001 |
| < 50 | Reference |  |  |  |
| 50–59 | 3.21 (1.82-5.66) | < 0.001 |  |  |
| 60–69 | 4.74 (2.49-9.03) | < 0.001 |  |  |
| 70–79 | 16.66 (6.22-44.62) | < 0.001 |  |  |
| Sex, male | 0.98 (0.62-1.56) | 0.944 |  |  |
| Cause of ESRD |  |  |  |  |
| Diabetes mellitus | 1.22 (0.89-1.75) | 0.451 |  |  |
| Hypertension | 0.80 (0.58-1.12) | 0.672 |  |  |
| Glomerulonephritis | 0.93 (0.52-2.15) | 0.591 |  |  |
| Cystic kidney disease | 1.19 (0.78-2.32) | 0.854 |  |  |
| Underlying disease |  |  |  |  |
| Cardiac disease |  |  |  |  |
| Coronary artery disease | 5.51 (2.74-11.06) | < 0.001 | 2.81 (1.37-5.78) | 0.005 |
| Acute myocardial infarction | 1.51 (0.37-6.15) | 0.566 |  |  |
| Cardiac arrhythmia | 2.53 (1.39-4.60) | 0.002 | 1.99 (1.09-3.64) | 0.025 |
| Cerebrovascular disease |  |  |  |  |
| Cerebral hemorrhage | NA |  |  |  |
| Cerebral infarction | 0.87 (0.12-6.26) | 0.890 |  |  |
| Hemodialysis | 3.00 (1.21-7.45) | 0.018 | 2.58 (1.04-6.42) | 0.041 |
| Dialysis vintage, months^c^ | 0.918 (0.833-1.012) | 0.086 |  |  |
| Before steroid use^d^ | 0.55 (0.17-1.74) | 0.307 |  |  |
| Infection |  |  |  |  |
| CMV infection | 1.26 (0.46-3.45) | 0.652 |  |  |
| Fungal infection | 0.66 (0.16-2.71) | 0.569 |  |  |
| Epoch of transplantation, 2010–2016 | 0.72 (0.45-1.15) | 0.168 |  |  |

^a^ Variables less than 0.05 of *P*-values in univariate analysis were included in the multivariate analysis.

^b^ NA is presented if the paucity of deceased or living patients exists for each variable 1 month after kidney transplantation.

^c^ Data were obtained from the Korean Network for Organ Sharing system.

^d^ The use of intravenous steroids such as dexamethasone, and prednisolone within 6 months before transplantation.Abbreviations: CI, confidence interval; CMV, cytomegalovirus; ESRD, end-stage renal disease; HR, hazard ratio; NA, not applicable.

**Table 3. Univariate and multivariate analyses of 3-month** mortality after kidney transplantation.

| Variable | Univariate | | Multivariate | |
| --- | --- | --- | --- | --- |
|  | HR (95% CI) | *P*-value | HR (95% CI) | *P*-value |
| Age, years^a^ | 1.07 (1.05–1.09) | < 0.001 | 1.08 (1.06-1.09) | < 0.001 |
| < 50 |  |  |  |  |
| 50–59 | 3.05 (2.09-4.44) | < 0.001 |  |  |
| 60–69 | 4.24 (2.74-6.57) | < 0.001 |  |  |
| 70–79 | 13.16 (6.43-26.96) | < 0.001 |  |  |
| Sex, female | 1.07 (0.78-1.46) | 0.690 |  |  |
| Cause of ESRD |  |  |  |  |
| Diabetes mellitus | 1.25 (0.92-1.59) | 0.273 |  |  |
| Hypertension | 0.86 (0.68-1.10) | 0.463 |  |  |
| Glomerulonephritis | 0.91 (0.49-1.75) | 0.385 |  |  |
| Cystic kidney disease | 1.23 (0.87-2.41) | 0.526 |  |  |
| Underlying disease |  |  |  |  |
| Cardiac disease |  |  |  |  |
| Coronary artery disease | 4.82 (2.92-7.97) | < 0.001 | 2.53 (1.49-4.31) | 0.001 |
| Acute myocardial infarction | 2.48 (1.16-5.29) | 0.019 | 1.70 (0.78-3.69) | 0.183 |
| Cardiac arrhythmia | 1.99 (1.28-3.10) | 0.002 | 1.43 (0.91-2.24) | 0.117 |
| Cerebrovascular disease |  |  |  |  |
| Cerebral hemorrhage | NA |  |  |  |
| Cerebral infarction | 0.80 (0.20-3.23) | 0.755 |  |  |
| Hemodialysis | 2.69 (1.49-4.85) | 0.001 | 2.32 (1.28-4.19) | 0.005 |
| Dialysis vintage, months^c^ | 0.963 (0.911-1.017) | 0.179 |  |  |
| Before steroid use^d^ | 0.95 (0.52-1.76) | 0.882 |  |  |
| Infection |  |  |  |  |
| CMV infection | 2.19 (1.51-3.16) | < 0.001 | 1.65 (1.12-2.42) | 0.012 |
| Fungal infection | 3.57 (2.47-5.15) | < 0.001 | 2.48 (1.69-3.65) | < 0.001 |
| Epoch of transplantation, 2010–2016 | 0.58 (0.42-0.79) | 0.001 | 0.43 (0.31-0.60) | < 0.001 |

^a^ Variables less than 0.05 of *P*-values in univariate analysis were included in the multivariate analysis.

^b^ NA is presented if the paucity of deceased or living patients exists for each variable 3 months after kidney transplantation.

^c^ Data were obtained from the Korean Network for Organ Sharing system.

^d^ The use of intravenous steroids such as dexamethasone, and prednisolone within 6 months before transplantation.

Abbreviations: CI, confidence interval; CMV, cytomegalovirus; ESRD, end-stage renal disease; HR, hazard ratio; NA, not applicable.

**Donor characteristics:**

Unfortunately, the donor characteristics were not provided by National Health Insurance Sharing Service. We have described this limitation in the revised Discussion section (page 18, lines 14 to 15) as follows.

“This study had several limitations. The lack of detailed clinical information, such as donor’s characteristics and laboratory data (immunologic antibody profiles, and serology for CMV and fungus), led to restrictions on the analysis of wider variables for TRM.”

**Comment 2**: In addition many topics need a better clarification and explanation: definition of CMD disease, prevalence of CMV serum-negativity. The cause of death classification is absolutely unreasonable, - “chronic kidney disease was the main cause of both early TRM and TRM, followed cystic kidney disease” ??????

**🡪 Response 2**: We have added explanation for the definition of CMV disease to the revised Materials Methods section (page 6, lines 16 to 17) as follows.

“The ICD-10-CM codes for CMV disease were B27.1, B25.0, B25.1, B25.8, and B25.9.”

The prevalence of CMV serum-negativity could not be provided because laboratory values for CMV infection were not included in the datasets provided by National Health Insurance Sharing Service. We have described this limitation in the revised Discussion section (page 18, lines 14 to 15) as follows.

“This study had several limitations. The lack of detailed clinical information, such as donor’s characteristics and laboratory data (immunologic antibody profiles, and serology for CMV and fungus), led to restrictions on the analysis of wider variables for TRM.”

We have eliminated the contents for the cause of death throughout the revised manuscript (Abstract, Materials and Methods, Results [Table 4, and S2 Table], and Discussion sections).

While revising your submission, please upload your figure files to the Preflight Analysis and Conversion Engine (PACE) digital diagnostic tool, https://pacev2.apexcovantage.com/. PACE helps ensure that figures meet PLOS requirements. To use PACE, you must first register as a user. Registration is free. Then, login and navigate to the UPLOAD tab, where you will find detailed instructions on how to use the tool. If you encounter any issues or have any questions when using PACE, please email us at figures@plos.org. Please note that Supporting Information files do not need this step.

- We have uploaded our figure files (Fig 1.tif, and Fig 2.tif) to the PACE digital diagnostic tool to meet PLOS requirements. The preview files (Preview_20200420051917202.pdf, and Preview_20200420052011519.pdf) were generated and checked.
