# Supplementary material for: The risk factors associated with treatment-related mortality in 16,073 kidney transplantation—A nationwide cohort study
Source: PLoS One. 2020 Jul 28;15(7):e0236274. doi: 10.1371/journal.pone.0236274 (PMC7386583; doi:10.1371/journal.pone.0236274)
Supplement: Attachment — Submitted filename: Response to Reviewers.doc [file pone.0236274.s003.doc]

**Response to the reviewer’s comments**

**Response to reviewer #1’s comments**

**1.** **I thank the authors to have addressed all my recommendations. At the moment the paper is suitable for publication.**

**🡪** We thank the reviewer for the constructive review of our manuscript.

**Response to reviewer #2’s comments**

**Treatment-related mortality (TRM) after renal transplantation is a concept different from disease-related mortality and appeared to be a very prevalent entity.
This article would be a valuable contribution to the medical literature to encourage further discussion on this entity.
The writing is clear and easily understandable.
The Authors have worked hard to improve this article, and they have met all criticisms raised by referees**
**Strengths:
- There are scarce data in scientific literature about TRM within 1 or 3 months after kidney transplantation.
- Authors collected an important amount of data from a very large cohort of patients using a national population based database, which included information about a total of 16,073 kidney recipients.
Specific comments:
1. Abstract. Authors should clearly explain the following sentence: “Based on a multivariate analysis, older age (hazard ratio [HR] = 1.07; P < 0.001), coronary artery disease (HR = 2.81; P = 0.005), and hemodialysis (HR = 2.58; P = 0.041) were the risk factors for early TRM.” What do you mean with hemodialysis as a risk factor for early TRM? Patients who underwent to hemodialysis immediately after renal transplant for a DGF, o hemodialysis compared with peritoneal dialysis or pre-emptive renal transplant? This is not clear.
🡪** We have provided detailed description of hemodialysis in the revised Abstract section as follows for clarification. In addition, we have also inserted this description into the revised Results section as follows.

**Abstract section** (page 3, lines 8 to 11)

“Based on a multivariate analysis, older age (hazard ratio [HR] = 1.06; *P* < 0.001), coronary artery disease (HR = 3.02; *P* = 0.002), and hemodialysis compared with pre-emptive kidney transplantation (HR = 2.53; *P =* 0.046) were the risk factors for early TRM.”

**Results section** (page 11, lines 22 to page 12, lines 2)

“Based on the Cox multivariate analysis, older age (hazard ratio [HR] = 1.06; *P* < 0.001), CAD (HR = 3.02; *P* = 0.002), cardiac arrhythmia (HR = 1.98; *P* = 0.027), and hemodialysis compared to pre-emptive kidney transplant (HR = 2.53; *P* = 0.046) were independently associated with early TRM.”

**2. Introduction, page 4, line 16. Supporting references at the end of the following sentence are needed: “however, studies about 1- or 3-month mortality were extremely limited.”**

**🡪** To the best of our knowledge, mortality after kidney transplantation within 1- or 3- months has been seldom reported. There was one report comparing mortality risk between deceased-donor kidney allograft recipients and wait-listed transplant candidates from time of listing published in 1993. Relative risk of mortality within the first 30 days, days 31-365 and more than 365-days post transplantation were 2.43, 0.96 and 0.36, respectively. Another report recently published was dealt with 30-day mortality and compared mostly the ethnic difference of England, and New York State. We have cited these two articles at the end of the sentence in the revised Introduction section as follows. These articles have been presented in the revised Reference section as follows.

**Introducton section** (page 4, lines 15 to 17)

“Most reports have shown the results of kidney transplantation after 1 [18], 5 [16], and greater than 10 years [19]; however, studies about 1- or 3-month mortality were extremely limited [20,21].”

**Reference section** (page 23, lines 20 to page 24, lines 1)

“20.	Port FK, Wolfe RA, Mauger EA, Berling DP, Jiang K. Comparison of survival probabilities for dialysis patients vs cadaveric renal transplant recipients. JAMA. 1993;270: 1339-1343.

21.	Tahir S, Gillott H, Jackson-Spence F, Nath J, Mytton J, Evison F, et al. Do outcomes after kidney transplantation differ for black patients in England versus New York State? A comparative, population-cohort analysis. BMJ Open. 2017;7: e014069.”

**3. Introduction, page 5, line 2. Authors should clearly explain the following sentence: “… to investigate the risk factors and causes of TRM after kidney transplantation focusing on vascular diseases.” Are Authors focused on vascular diseases in this analysis?**

**🡪** As indicated by the reviewer, various risk factors related to treatment-related mortality (TRM) were identified in our manuscript. Therefore, we have eliminated “focusing on vascular diseases” in the revised Introduction section (page 5, lines 1 to 2) as follows.

“Using this database, we performed a comprehensive population-based analysis to investigate the risk factors and causes of TRM after kidney transplantation.”

**4. Methods. Authors collected data of the post-transplant administration of antiviral agent, but there is no mention to induction (basiliximab vs thymoglobuline) immunosuppressive therapy, that is supposed to strongly impact on treatment related mortality. This is an important point that Authors should add into the analysis. Otherwise this will represent an important limitation.**

**🡪** As suggested by the reviewer, we have extracted the information about induction regimens and provided the results, and discussion in the revised manuscript. Because the recipients receiving anti-thymocyte globulin were significantly associated with 1- and 3-month mortalities in the multivariate analyses, the results of other variables such as age, coronary artery disease, cardiac arrhythmia, and hemodialysis in the early TRM, and age, coronary artery disease, acute myocardial infarction, cardiac arrhythmia, hemodialysis, and cytomegalovirus and fungal infections in TRM were also revised throughout the manuscript. In particular, the contents for CMV infection related to TRM including Fig 2B were eliminated because the *P* value was changed from 0.012 to 0.106 in the revised manuscript.

**Abstract section** (page 3, lines 8 to 15)

“Based on a multivariate analysis, older age (hazard ratio [HR] = 1.06; *P* < 0.001), coronary artery disease (HR = 3.02; *P* = 0.002), and hemodialysis compared with pre-emptive kidney transplantation (HR = 2.53; *P =* 0.046) were the risk factors for early TRM. Older age (HR = 1.07; *P* < 0.001), coronary artery disease (HR = 2.88; *P* < 0.001), and hemodialysis (HR = 2.35; *P* = 0.004) were the common independent risk factors for TRM. In contrast, cardiac arrhythmia (HR = 1.98; *P* = 0.027) was associated only with early TRM, and fungal infection (HR = 2.61; *P* < 0.001), and epoch of transplantation (HR = 0.34; *P <* 0.001) were the factors associated with only TRM.”

**Materials and Methods section** (page 6, line 15)

“The induction regimens such as basiliximab, and anti-thymocyte globulin were also extracted.”

**Results section** (page 8, lines 16 to 17)

“Regarding to induction therapy, basiliximab, and anti-thymocyte globulin were administered to 79.0%, and 11.4% of recipients, respectively.”

**Results section** (page 11, lines 16 to 18)

“Patients with anti-thymocyte globulin showed significant relation to TRM (*P* < 0.001), whereas those with basiliximab did not.”

**Results section** (page 11, lines 22 to page 12, lines 5)

“The risk factors of early TRM and TRM are shown in Tables 2 and 3, respectively. Based on the Cox multivariate analysis, older age (hazard ratio [HR] = 1.06; *P* < 0.001), CAD (HR = 3.02; *P* = 0.002), cardiac arrhythmia (HR = 1.98; *P* = 0.027), and hemodialysis compared to pre-emptive kidney transplant (HR = 2.53; *P* = 0.046) were independently associated with early TRM. Moreover, older age (HR = 1.07; *P* < 0.001), CAD (HR = 2.88, *P* = < 0.001), and hemodialysis (HR = 2.35, *P* = 0.004) were consistently independent risk factors of TRM at any time. However, fungal infection, (HR = 2.61; *P* < 0.001), and the epoch of transplantation (HR = 0.34 for 2010–2016; *P* < 0.001) were correlated to TRM only.”

**Results section** (page 15, lines 10 to 11)

“Fungal infection (Fig 2B) affected TRM (after early TRM). The protective effect of transplantation in 2010–2016 is illustrated in Fig 2C.”

**Discussion section** (page 17, lines 8 to 19)

“The use of anti-thymocyte globulin has been greater in high-risk recipients such as highly sensitized patients, recipients from deceased donors, re-transplantations, and ABO incompatible transplants [36]. According to a prospective, randomized study, patients receiving anti-thymocyte globulin presented a greater incidence of infection (85.8%) compared to those with basiliximab (75.2%) at 12 months after transplantation [37]. However, there was no significant difference in patient survival, similar to the results of a recent study using a network meta-analysis [38]. In Korea, the one-year patient survival in the anti-thymocyte globulin group (89.4%) was compared to the basiliximab group (93.8%), and presented no significant difference [39]. Based on our data, the high-risk recipients receiving anti-thymocyte globulin were significantly associated with early mortality. Further studies for the premature mortality are necessary to validate our results, and intensive care for the high-risk patients receiving anti-thymocyte globulin is important for improving outcomes.”

**Figure** (page 15, lines 18 to 23)

**Fig 2. Cumulative incidence of mortality according to the factors associated 1- or 3-month mortality after kidney transplantation.**

(A) Cardiac arrhythmia was related to a worse outcome 1 month after transplantation. (B) Fungal infection were a risk factor of 3-month mortality after transplantation. (C) Recent epoch of transplantation (2010–2016) was a protective factor of 3-month mortality compared to the treatment-related mortality of previous epoch (2003–2009).

Table 1. Comparison of the characteristics between living kidney recipients versus deceased ones at 1 and 3 months after transplantation.

| **Characteristics^a^** | **Early TRM** | | | **TRM** | | |
| --- | --- | --- | --- | --- | --- | --- |
|  | **Living at 1 month** | **Death by 1 month** | ***P*-value^b^** | **Living at 3 months** | **Death by 3 months** | ***P*-value^b^** |
| Number (%) | 15,999 | 74 |  | 15,913 | 160 |  |
| Age, years | 47 (37-55) | 56 (48.8-61) | < 0.001 | 47 (37-55) | 55.5 (48-61) | < 0.001 |
| < 50 | 9,188 (57.4) | 19 (25.7) | < 0.001 | 9,163 (57.6) | 44 (27.5) | < 0.001 |
| 50–59 | 4,830 (30.2) | 32 (43.2) |  | 4,792 (30.1) | 70 (43.8) |  |
| 60–69 | 1,838 (11.5) | 18 (24.3) |  | 1,819 (11.4) | 37 (23.1) |  |
| 70–79 | 143 (0.9) | 5 (6.8) |  | 139 (0.9) | 9 (5.6) |  |
| Sex, male | 9,451 (59.1) | 44 (59.5) | 0.946 | 9,403 (59.1) | 92 (57.5) | 0.684 |
| Cause of ESRD |  |  |  |  |  |  |
| Diabetes mellitus | 3,501 (21.9) | 19 (25.7) | 0.431 | 3,479 (21.9) | 41 (25.6) | 0.252 |
| Hypertension | 2,001 (12.5) | 8 (10.8) | 0.660 | 1,992 (12.5) | 17 (10.6) | 0.471 |
| Glomerulonephritis | 2,850 (17.8) | 11 (14.9) | 0.508 | 2,840 (17.8) | 21 (13.1) | 0.120 |
| Cystic kidney disease | 368 (2.3) | 2 (2.7) | 0.818 | 365 (2.3) | 5 (3.1) | 0.485 |
| Underlying disease^c^ |  |  |  |  |  |  |
| Cardiac disease |  |  |  |  |  |  |
| Coronary artery disease | 392 (2.5) | 9 (12.2) | < 0.001 | 384 (2.4) | 17 (10.6) | < 0.001 |
| Acute myocardial infarction | 288 (1.8) | 2 (2.7) | 0.561 | 283 (1.8) | 7 (4.4) | 0.014 |
| Cardiac arrhythmia | 1,240 (7.8) | 13 (17.6) | 0.002 | 1,230 (7.7) | 23 (14.4) | 0.002 |
| Cerebrovascular disease |  |  |  |  |  |  |
| Cerebral hemorrhage | 54 (0.3) | 0 (0.0) | 0.617 | 54 (0.3) | 0 (0.0) | 0.460 |
| Cerebral infarction | 247 (1.5) | 1 (1.4) | 0.893 | 246 (1.5) | 2 (1.3) | 0.763 |
| Hemodialysis | 13,134 (82.1) | 69 (93.2) | 0.012 | 13,055 (82.0) | 148 (92.5) | 0.001 |
| Dialysis vintage, months^d^ | 42.5 (29.5-62.8) | 16.0 (9.5-24.5) | 0.051 | 41.0 (29.0-63.5) | 24.5 (12.8-39.0) | 0.179 |
| Before steroid use^e^ | 1,148 (7.2) | 3 (4.1) | 0.416 | 1,140 (7.2) | 11 (6.9) | 1.000 |
| Induction therapy |  |  |  |  |  |  |
| Basiliximab | 12,637 (79.0) | 55 (74.3) | 0.402 | 12,569 (79.0) | 123 (76.9) | 0.579 |
| Anti-thymocyte globulin | 1,818 (11.4) | 22 (29.7) | < 0.001 | 1,799 (11.3) | 41 (25.6) | < 0.001 |
| Infection |  |  |  |  |  |  |
| CMV infection | 694 (4.3) | 4 (5.4) | 0.653 | 1,900 (11.9) | 37 (23.1) | < 0.001 |
| Fungal infection | 639 (4.0) | 2 (2.7) | 0.571 | 1,205 (7.6) | 37 (23.1) | < 0.001 |
| Epoch of transplantation |  |  |  |  |  |  |
| 2003–2009 | 4,634 (29.0) | 27 (36.5) | 0.155 | 4,594 (28.9) | 67 (41.9) | < 0.001 |
| 2010–2016 | 11,365 (71.0) | 47 (63.5) |  | 11,319 (71.1) | 93 (58.1) |  |

^a^ Data were expressed as number (%) or median (interquartile range).

^b^ *P* value was calculated using chi-square test or Mann–Whitney U test.

^c^ In case of the presence of underlying diseases, multiple diseases were designated to one patient.

^d^ Data were obtained from the Korean Network for Organ Sharing system.

^e^ The use of intravenous steroids such as dexamethasone, and prednisolone within 6 months before transplantation.

Abbreviations: CMV, cytomegalovirus; ESRD, end-stage renal disease; TRM, treatment-related mortality.

Table 2. Univariate and multivariate analyses of 1-month mortality after kidney transplantation.

| Variable | Univariate | | Multivariate | |
| --- | --- | --- | --- | --- |
|  | HR (95% CI) | *P*-value | HR (95% CI) | *P*-value |
| Age, years^a^ | 1.07 (1.05–1.10) | < 0.001 | 1.06 (1.04-1.09) | < 0.001 |
| < 50 | Reference |  |  |  |
| 50–59 | 3.21 (1.82-5.66) | < 0.001 |  |  |
| 60–69 | 4.74 (2.49-9.03) | < 0.001 |  |  |
| 70–79 | 16.66 (6.22-44.62) | < 0.001 |  |  |
| Sex, male | 0.98 (0.62-1.56) | 0.944 |  |  |
| Cause of ESRD |  |  |  |  |
| Diabetes mellitus | 1.22 (0.89-1.75) | 0.451 |  |  |
| Hypertension | 0.80 (0.58-1.12) | 0.672 |  |  |
| Glomerulonephritis | 0.93 (0.52-2.15) | 0.591 |  |  |
| Cystic kidney disease | 1.19 (0.78-2.32) | 0.854 |  |  |
| Underlying disease |  |  |  |  |
| Cardiac disease |  |  |  |  |
| Coronary artery disease | 5.51 (2.74-11.06) | < 0.001 | 3.02 (1.48-6.17) | 0.002 |
| Acute myocardial infarction | 1.51 (0.37-6.15) | 0.566 |  |  |
| Cardiac arrhythmia | 2.53 (1.39-4.60) | 0.002 | 1.98 (1.08-3.62) | 0.027 |
| Cerebrovascular disease |  |  |  |  |
| Cerebral hemorrhage | NA |  |  |  |
| Cerebral infarction | 0.87 (0.12-6.26) | 0.890 |  |  |
| Hemodialysis | 3.00 (1.21-7.45) | 0.018 | 2.53 (1.02-6.28) | 0.046 |
| Dialysis vintage, months^c^ | 0.918 (0.833-1.012) | 0.086 |  |  |
| Before steroid use^d^ | 0.55 (0.17-1.74) | 0.307 |  |  |
| Induction therapy |  |  |  |  |
| Basiliximab | 0.77 (0.46-1.30) | 0.326 |  |  |
| Anti-thymocyte globulin | 3.31 (2.01-5.45) | < 0.001 | 2.62 (1.59-4.32) | < 0.001 |
| Infection |  |  |  |  |
| CMV infection | 1.26 (0.46-3.45) | 0.652 |  |  |
| Fungal infection | 0.66 (0.16-2.71) | 0.569 |  |  |
| Epoch of transplantation, 2010–2016 | 0.72 (0.45-1.15) | 0.168 |  |  |

^a^ Variables less than 0.05 of *P*-values in univariate analysis were included in the multivariate analysis.

^b^ NA is presented if the paucity of deceased or living patients exists for each variable 1 month after kidney transplantation.

^c^ Data were obtained from the Korean Network for Organ Sharing system.

^d^ The use of intravenous steroids such as dexamethasone, and prednisolone within 6 months before transplantation.

Abbreviations: CI, confidence interval; CMV, cytomegalovirus; ESRD, end-stage renal disease; HR, hazard ratio; NA, not applicable.

Table 3. Univariate and multivariate analyses of 3-month mortality after kidney transplantation.

| Variable | Univariate | | Multivariate | |
| --- | --- | --- | --- | --- |
|  | HR (95% CI) | *P*-value | HR (95% CI) | *P*-value |
| Age, years^a^ | 1.07 (1.05–1.09) | < 0.001 | 1.07 (1.05-1.09) | < 0.001 |
| < 50 |  |  |  |  |
| 50–59 | 3.05 (2.09-4.44) | < 0.001 |  |  |
| 60–69 | 4.24 (2.74-6.57) | < 0.001 |  |  |
| 70–79 | 13.16 (6.43-26.96) | < 0.001 |  |  |
| Sex, female | 1.07 (0.78-1.46) | 0.690 |  |  |
| Cause of ESRD |  |  |  |  |
| Diabetes mellitus | 1.25 (0.92-1.59) | 0.273 |  |  |
| Hypertension | 0.86 (0.68-1.10) | 0.463 |  |  |
| Glomerulonephritis | 0.91 (0.49-1.75) | 0.385 |  |  |
| Cystic kidney disease | 1.23 (0.87-2.41) | 0.526 |  |  |
| Underlying disease |  |  |  |  |
| Cardiac disease |  |  |  |  |
| Coronary artery disease | 4.82 (2.92-7.97) | < 0.001 | 2.88 (1.71-4.84) | < 0.001 |
| Acute myocardial infarction | 2.48 (1.16-5.29) | 0.019 | 1.75 (0.81-3.80) | 0.157 |
| Cardiac arrhythmia | 1.99 (1.28-3.10) | 0.002 | 1.40 (0.89-2.18) | 0.145 |
| Cerebrovascular disease |  |  |  |  |
| Cerebral hemorrhage | NA |  |  |  |
| Cerebral infarction | 0.80 (0.20-3.23) | 0.755 |  |  |
| Hemodialysis | 2.69 (1.49-4.85) | 0.001 | 2.35 (1.30-4.25) | 0.004 |
| Dialysis vintage, months^c^ | 0.963 (0.911-1.017) | 0.179 |  |  |
| Before steroid use^d^ | 0.95 (0.52-1.76) | 0.882 |  |  |
| Induction therapy |  |  |  |  |
| Basiliximab | 0.88 (0.61-1.28) | 0.514 |  |  |
| Anti-thymocyte globulin | 2.73 (1.92-3.90) | < 0.001 | 2.38 (1.62-3.49) | < 0.001 |
| Infection |  |  |  |  |
| CMV infection | 2.19 (1.51-3.16) | < 0.001 | 1.39 (0.93-2.08) | 0.106 |
| Fungal infection | 3.57 (2.47-5.15) | < 0.001 | 2.61 (1.79-3.82) | < 0.001 |
| Epoch of transplantation, 2010–2016 | 0.58 (0.42-0.79) | 0.001 | 0.34 (0.24-0.48) | < 0.001 |

^a^ Variables less than 0.05 of *P*-values in univariate analysis were included in the multivariate analysis.

^b^ NA is presented if the paucity of deceased or living patients exists for each variable 3 months after kidney transplantation.

^c^ Data were obtained from the Korean Network for Organ Sharing system.

^d^ The use of intravenous steroids such as dexamethasone, and prednisolone within 6 months before transplantation.

Abbreviations: CI, confidence interval; CMV, cytomegalovirus; ESRD, end-stage renal disease; HR, hazard ratio; NA, not applicable.

**5. Results. A sub-analysis might be performed distinguishing TRM analysis between transplantation cases performed in different epoch (2003-2009 vs 2010-2016). This might be interesting even if this factor did not reach statistical significance at univariate analysis. In fact, “Epoch of transplantation 2010–2016” showed a trend towards statistical significance.**

🡪 We have conducted the sub-analysis for the two epoch of transplantation and added the results to the revised Results and Discussion sections as follows.

**Results section** (page 12, lines 6 to 10)

“Regarding to the epoch of transplantation, the aged between 50 and 59 years (HR = 0.37, *P* = 0.005 for early TRM; HR = 0.37, *P* < 0.001 for TRM), the patients receiving basiliximab as induction therapy (HR = 0.44, *P* = 0.002 for early TRM; HR = 0.40, *P* < 0.001 for TRM), and recipients with CMV infection (HR = 0.13, *P* = 0.040 for early TRM; HR = 0.39, *P* = 0.005 for TRM) presented better outcome in 2010-2016, when compared to 2003-2009.”

**Discussion section** (page 18, lines 22 to page 19, lines 4)

“In particular, relatively low- or intermediate-risk patients such as aged 50 to 59 years, and patients receiving basiliximab were influence by the improved protocols, and showed better outcome than high-risk recipients (aged over 60 years, and recipients with anti-thymocyte globulin). Further, more aggressive and sophisticated infection controls on CMV such as monitoring quantitative levels, and high dose of antiviral therapy [47] may protect more recipients in 2010-2016 than those in 2003-2009.”

**6. Results. In the following sentence, Authors should specify the number of recipients who died among the total number of recipients for each type of transplantation, and only after the percentage value in parentheses: “The rates of recipients who died 1 month (1.2% for living, 2.4% for deceased, and
3.3% for non-heart beating) and 3 months (3.2% for living, 5.5% for deceased, and 3.3% for non-heart beating) after transplantation showed significant difference according to the donor state (*P* < 0.001).”**

**🡪** We have applied these results to our cohort and presented the number of recipients in the revised Results section (page 11, lines 9 to 12) as follows. The calculation errors were also corrected.

“The rates of recipients who died 1 month (n = 1, 1.4% for living; n= 2, 2.7% for deceased; and n = 2, 2.7% for non-heart beating) and 3 months (n = 5, 3.1% for living; n = 9, 5.6% for deceased; and n = 5, 3.1% for non-heart beating) after transplantation showed significant difference according to the donor state (*P* < 0.001).”

**Response to the editor’s comment**

While revising your submission, please upload your figure files to the Preflight Analysis and Conversion Engine (PACE) digital diagnostic tool, https://pacev2.apexcovantage.com/. PACE helps ensure that figures meet PLOS requirements. To use PACE, you must first register as a user. Registration is free. Then, login and navigate to the UPLOAD tab, where you will find detailed instructions on how to use the tool. If you encounter any issues or have any questions when using PACE, please email us at figures@plos.org. Please note that Supporting Information files do not need this step.

- We have uploaded our revised figure files (Fig 1.tif, and Fig 2.tif) to the PACE digital diagnostic tool to meet PLOS requirements. The preview files (Preview_20200623002121848.pdf, and Preview_20200623002149051.pdf) were generated and checked.
